# Comparative Histology and Histochemistry of the Parotid Gland and Mandibular Gland in the Lowland Tapir (*Tapirus terrestris* Perissodactyla) and Aardvark (*Orycteropus afer* Tubulidentata)

**DOI:** 10.3390/ani13101684

**Published:** 2023-05-18

**Authors:** Joanna Klećkowska-Nawrot, Karolina Barszcz, Jan Paweł Miniajluk, Oleksii Melnyk, Karolina Goździewska-Harłajczuk

**Affiliations:** 1 Department of Biostructure and Animal Physiology, Wrocław University of Environmental and Life Sciences, Kozuchowska 1, 51-631 Wrocław, Poland; 2 Department of Morphological Sciences, Institute of Veterinary Medicine, Warsaw University of Life Sciences–SGGW, Nowoursynowska 159c, 02-776 Warsaw, Poland; karolina_barszcz@sggw.edu.pl; 3 Faculty of Veterinary Medicine, “Alkmeon” Anatomy Scientific Society, Wroclaw University of Environmental and Life Sciences, Kozuchowska 1, 51-631 Wroclaw, Poland; 123375@student.upwr.edu.pl; 4 Department of Animal Anatomy, Histology and Pathomorphology, National University of Life and Environmental Sciences of Ukraine, Heroiv Oborony Str. 15, 03041 Kyiv, Ukraine; melnik_oo@nubip.edu.ua

**Keywords:** aardvark, glands of oral cavity, lowland tapir, major salivary glands, mandibular gland, parotid gland

## Abstract

**Simple Summary:**

The goal of this study was to perform a comparative analysis of the microstructure and secretion of the parotid and mandibular glands of adult female lowland tapir and aardvark. Histological and histochemical analyses showed that while the parotid gland of the lowland tapir consists of numerous large lobes of different shapes, those of the aardvark were smaller and more ovoid in shape. The parotid gland lobes were of different sizes (small, medium, and large) and shapes in the tapir, while all were roughly ovoid in shape in the aardvark. The obtained results showed that the mandibular gland secretion had a mucoserous character in aardvark, while in lowland tapir, the dominance of mucous secretion was determined. These differences could be related to the specific diets of these two different species.

**Abstract:**

In terrestrial mammals, the parotid and mandibular glands secrete different types of saliva into the oral cavity. Both glands were obtained from two female lowland tapirs (*Tapirus terrestris*) and one female aardvark (*Orycteropus afer*) from the Wroclaw Zoological Garden (Poland) and examined by light microscopy (hematoxylin and eosin, mucicarmine, periodic acid-Schiff, Alcian blue pH 1.0, Alcian blue pH 2.5, Alcian blue pH 2.5/PAS, and Hale’s dialysed iron). Both the parotid glands observed in the lowland tapir and aardvark were compound alveolar serous secretory units, and in both species, the secretion was composed of neutral and acidic mucopolysaccharides (sialo and sulfated mucins). However, in both the lowland tapir and aardvark, a histological examination found the stroma of the mandibular gland was divided into very large lobes by poorly marked connective tissue. While many interlobar and striated ducts were found in the aardvark, very few were found in the lowland tapir. The mandibular gland was a branched tubular (mucous secretion) type in the lowland tapir, but it was a branched tubuloalveolar (mucous-serous) type in the aardvark. In all tested glands, the secretion was composed of neutral mucopolysaccharides, acid-sulfated mucosubstances, and sialomucins.

## 1. Introduction

The glands of the oral cavity (*glandulae oris*) can be divided into minor salivary glands (*glandulae salivariae minores*) located in the mucous and submucous membranes of the oral cavity, and major salivary glands (*glandulae salivarie majores*) located outside the wall of the oral cavity [1,2,3,4]. Major salivary glands (mandibular, parotid, monostomatic sublingual, and polystomatic sublingual glands) and zygomatic glands (i.e., dorsal buccal glands located in the *regio zygomatica* according to *NAV* [2] are classified as minor salivary glands) deliver their secretions through excretory ducts, leading into the oral vestibule and oral cavity proper [1,2,3,4]. The parotid gland of domestic mammals is the largest of the major salivary glands, located at the base of the auricle, between the branch of the lower jaw and the wing of the atlas, in *fossa retromandibularis* [1,3]. However, in domestic animals, the mandibular gland lies under the body of the mandible, between the wing of the atlas and the basihyoideum [1,3]. 

The secretions of the major and minor salivary glands play various important roles: they facilitate the intake of food and moisturize food, making it easier to swallow and providing lubrication for vocalization. The enzyme contained in saliva, salivary amylase, is responsible for the initial stage of digesting sugar, and bactericidal substances (lysozyme, lactoferrin, and sialoperoxidase system) limit the growth of bacteria in the oral cavity, protecting against harmful pathogens. In addition, carbonate and phosphate ions help maintain the correct pH in the oral cavity. Saliva is also rich in other ions (including calcium and phosphates) responsible for maintaining the balance between demineralization and remineralization, which play important roles in caries formation [5,6,7,8,9,10,11,12]. In addition, saliva is involved in the immunological response through IgA and plays a role in secreting potassium and resorbing sodium [13,14,15,16,17]. 

In animals, both diet and habitat influence the size of the glands of the oral cavity, the structure of secretory units, and the nature of their secretion (serous, mucous, or mixed). For example, in herbivores, the parotid gland is larger, and the serous parotid secretion is more copious [1,18,19,20,21]. 

The lowland tapir which belongs to the same Perissodactyla order as the Rhinocerotidae and Equidae, is a special herbivore. Although it prefers seasonal and locally available plant species and various plant parts (saplings, ferns, vines, palms, trees and their roots, flowers, bark, twigs, leaves, and stems), it may also supplement its diet with fruits, especially *Spondias mombin*, *Helicostylis tomentosa*, *Ficus* spp., and *Bagassa guianensis*, depending on the availability of food resources [22,23,24,25,26,27]. It particularly prefers the soft terminal and the youngest parts of plants, as these are more nutritious and less fibrous [25,28,29]. The first structural description of the parotid gland and mandibular gland (as both glands) well developed in the tapir (species name not given) was described by Turner in 1850 [30] (cited by Padilla and Dowler [31]) and by Quintarelli and Dellovo [32]. 

In contrast, the aardvark mainly feeds on termites (*Trinervitermes*, *Cubitermes*, *Macrotermes*, *Hodotermes*, *Odontotermes*, *Microtermes*, and *Pseudacanthotermes*), ants (*Anoplepis*, *Camponotus*, *Crematogaster*, and *Dorylusand*), grasshoppers (*Saltotoria*), beetles (pupae and larvae) (*Coleptera*), especially of the Scarabidae family [33,34,35,36,37], and the only fruit it easts is the cucurbit Cucumis humifructus [34,38]. The first histological description of the parotid and mandibular glands in the aardvark was presented by Quintarelli and Dellovo in 1969 [32]. The aardvark engages in feeding behavior known as myrmecophagy, similar to inter alia the pangolin (*Manis* spp.), giant anteater (*Myrmecophaga tridactyla*), dwarf anteater (*Cyclopes didactylus*), armadillo (*Dasypus* spp.), aardwolf (*Proteles cristatus*), cape fox (*Vulpes chama*), numbat (*Myrmecobius fasciatus*), echidna (*Zaglossus* and *Tachyglossus*), honey badger (*Mellivora capensis*), jackal (*Canis* spp.), yellow mongoose (*Cynictis penicullata*), small-spotted genet (*Genetta genetta*), striped polecat (*Ictonyx striatus*), southern tamandua (Tamandua tetradactyla), and sloth bear (*Melursus ursinus*) [34,39]. 

The morphology of the major salivary glands has been described in some myrmecophagic animals (echidna *Tachyglossus aceleatus*; southern long-nosed armadillo *Dasypus hybridus*; Malayan pangolin *Manis javanica*; Chinese pangolin *Manis pentadactyla*; aardwolf; southern tamandua; giant anteater; big hairy armadillo *Chaetophractus villosus*, screaming hairy armadillo *Chaetophractus vellerosus*, nine-banded armadillo *Dasypus novemcinctus*, and pichi *Zaedyus pichiy*) [40,41,42,43,44,45,46,47,48,49,50,51,52,53,54,55,56,57] and some species from Perissodactyla (*Ceratotherium* spp., *Diceros* spp., *Rhinoceros* spp., donkey, and horse) [1,3,58,59,60,61,62,63,64,65].

The present paper examines the histological structures and compositions of the glandular secretions of the parotid gland and mandibular gland of the lowland tapir and aardvark, and compares the findings with those of Quintarelli and Dellovo from 1969 [32], as well as with observations based on other myrmecophagous mammals and Perissodactyla. 

## 2. Materials and Methods

### 2.1. Animals

Two adult female lowland tapirs and one adult female aardvark from the Wroclaw Zoological Garden (Wrocław, Poland) were examined. The animals were collected in 2019 and 2021 (tapirs) and 2017 (aardvark) in the Division of Animal Anatomy, Wroclaw University of Environmental and Life Sciences. The age of the examined animals on the day of death was as follows: the first female lowland tapir was 25 years, three months and 27 days old; the second female lowland tapir was 31 years, one month and two days old; the female aardvark was five years, seven months and 16 days old. 

### 2.2. Tissue Samples

During the post-mortem examination, three pairs of parotid glands and three pairs of the mandibular gland were taken. Samples for histological examination were collected in consecutive order: 4 samples from the left parotid gland and 4 samples from the right parotid gland of the aardvark; 4 samples from the left mandibular gland and 4 samples from the right mandibular gland of the aardvark; 8 samples from the left parotid gland and 8 samples from the right parotid gland from both tapirs; 8 samples from the left mandibular gland and 8 samples from the right mandibular gland from both tapirs. These samples were fixed directly in 4% buffered formaldehyde for at least 72 h and then dehydrated using 75%, 96%, and 100% ethanol. The samples were processed in an ETP vacuum tissue processor (RVG3, Intelsint, Villarbasse, Italy), embedded in paraffin blocks, and cut into 4 µm sections using a Slide 2003 (Pfm A.g., Köln, Germany) sliding microtome. The samples were stained with hematoxylin, eosin, and mucicarmine, as previously described by Burck [66]. In addition, the periodic acid-Schiff; Alcian blue pH 1.0; Alcian blue pH 2.5; Alcian blue pH 2.5/PAS, and Hale’s dialysed iron methods were used to evaluate the composition of the glandular secretion [67,68,69,70,71]. The slides were analyzed using a Zeiss Axio Scope A1 light microscope (Carl Zeiss, Jena, Germany). The histochemical evaluation of the parotid and mandibular glands samples in the three animals was performed according to Spicer and Henson [71], where (−) indicated a negative reaction; (−/+) and (+) a weak reaction; (++) a mild reaction, and (+++) a strong reaction. The histological description of the examined structure of the parotid gland and mandibular gland was based on NAH [72]. The histological measurements of the outer diameter of the secretion units were using with the Axio Vision Rel. 4.8 Software—Carl Zeiss.

### 2.3. Ethical Statement

The examined lowland tapirs and aardvarks were not killed for the purpose of this study and died under natural circumstances. Personal permits (for post-mortem collection) were obtained from the Country Veterinary Officer in Wroclaw (Poland) (No. PIW Wroc. UT-45/5/16—Dr. Joanna Klećkowska-Nawrot; No. PIW Wroc. UT-45/6/16—Dr Karolina Goździewska-Harłajczuk). According to Polish law and European law, studies on tissues obtained post-mortem do not require the approval of the Ethics Committee (*Journal of Laws of the Republic of Poland*, the Act of 15 January 2015, on the protection of animals used for scientific or educational purposes; European Parliament and Council Directive 2010/63/UE dated 22 September 2010 regarding the protection of animals used for scientific purposes).

## 3. Results

### 3.1. Parotid Gland Histology and Histochemistry

**Figure 1:**
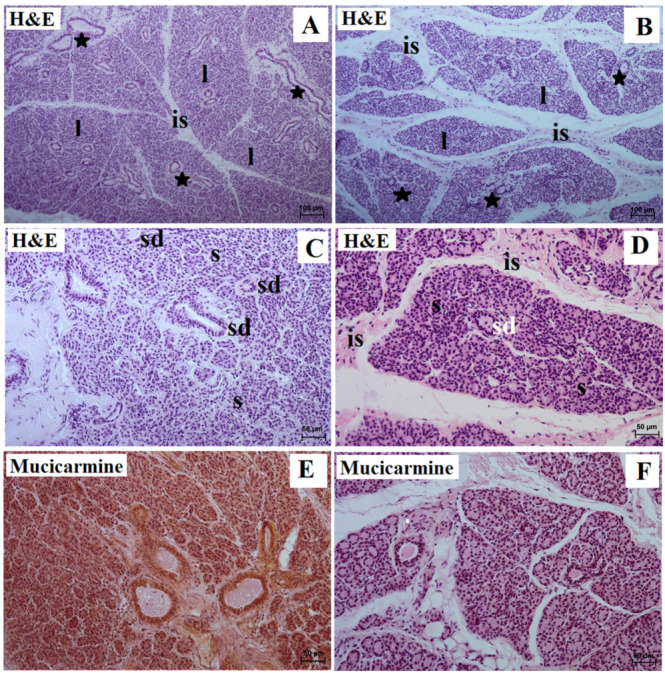
Photomicrograph of the lowland tapir (**A**,**C**,**E**) and aardvark (**B**,**D**,**F**) parotid’s gland showing serous acini (s) and excretory interlobar ducts (asterisk), which were lined with cuboidal epithelium (**A**,**B**). The striated ducts (sd) consisted of pyramidal cells with basal striations (**C**,**D**). The mucicarmine stain confirms a negative reaction (−) of the lowland tapir serous acini and a slight reaction (+) of the aardvark serous acini. is—interlobar septa; l—lobes. Bar = 100 µm (**A**,**B**); 50 µm (**C**–**F**).

In the examined animals, the parotid gland was surrounded by a thin connective tissue capsule made of loose fibrous connective tissue. In the lowland tapir, the stroma of the gland was formed by the septa consisting of thin connective tissue, divided into numerous lobes; in the aardvark, thick partitions were present, which divided the tissue into a large number of small lobes (Figure 1A,B, Table 1). While the lobes were of different sizes (small, medium, and large) and shapes (oval, elongated, triangular, and quadrangular) in the tapir, all were roughly ovoid in shape in the aardvark (Figure 1A,B, Table 1). 

The interlobar septa of the gland presented excretory interlobar ducts, blood vessels, and nerves. The interlobar ducts in the examined animals were composed of taller columnar cells and exhibited central or basally located oval or round nuclei: these were present in high numbers in the interlobar septa of the tapir but were much fewer in the aardvark (Figure 1A,B, Table 1). The vast majority of the interlobar ducts in the lowland tapir were broad with wide lumina, while those in the aardvark were small with narrow lumina (Figure 1A,B, Table 1). 

In both the lowland tapir and aardvark, the parotid glands were branched alveolar complexes, producing serous secretions. The secretory endpieces consisted of single conical cells, characterized by basally positioned and oval or spherical nuclei. The mean outer diameter of the acini was 36.0 µm in the lowland tapir and 25.0 µm in the aardvark. The secretory part empties into an intercalated duct, which then leads to striated ducts located between the secretory units (Figure 1C,D, Table 1). The intercalated duct was composed of cuboidal cells; however, the striated ducts were characterized by cuboidal or columnar cells containing basal striations. While many striated ducts were noted in the lowland tapir, few were observed in the aardvark. The myoepithelial cells appeared to be elongated and spindle in shape and were observed on the external surface of the alveolar secretory units. 

The mucicarmine stain was negative (−) in the secretory units and ducts of the tapir, while a negative reaction (−) was observed in the acini, striated ducts, and interlobar ducts of the aardvark (Figure 1E,F, Table 1).

**Figure 2:**
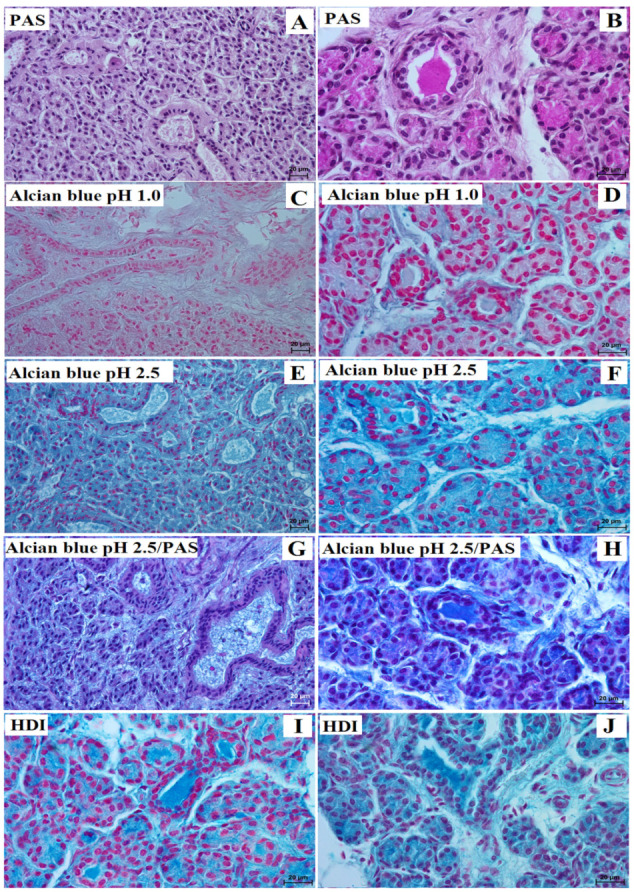
Photomicrograph of the lowland tapir (**A**,**C**,**E**,**G**,**I**) and aardvark (**B**,**D**,**F**,**H**,**J**) histochemistry parotid’s gland. Alcian blue pH 1.0 showed a negative reaction (−) in both species (**C**,**D**) and HDI showed a negative reaction (−) only in the lowland tapir (**I**). The secretion of the lowland tapir and aardvark was composed of PAS-positive neutral and Alcian blue pH 2.5, Alcian blue pH 2.5/PAS (magenta in aardvark; blue in lowland tapir), and HDI (aardvark) positive acid mucopolysaccharides (sulfated and sialomucins) (**A**,**B**,**E**–**H**,**J**). Bar = 20 µm (**A**–**J**).

Regarding the PAS stain, a negative (−) reaction was observed in the acini of the lowland tapir, while a strongly positive (+++) PAS and HDI reaction was observed in the aardvark (Figure 2A,B,I,J, Table 1). The interlobar ducts were mostly strongly (+++) PAS positive in both animals, while the striated ducts were strongly (+++) PAS positive in the aardvark but slightly (−/+) PAS reactive in the tapir (Figure 2A,B, Table 1). 

The Alcian blue pH 1.0 stain was negative (−) in the acini and interlobar ducts and striated ducts in both the lowland tapir and aardvark (Figure 2C,D). In addition, the Alcian blue pH 2.5 stain was found to be strongly (+++) positive in the acini, interlobar and striated ducts in both animals (Figure 2E,F, Table 1). The secretion yielded a strongly positive (+++) Alcian blue pH 2.5/PAS-positive reaction in both the tapir and aardvark: a magenta color in the aardvark, but a blue color in the tapir (Figure 2G,H, Table 1). 

The HDI stain yielded a strongly (+++) positive reaction in the system ducts of the aardvark’s parotid gland (Figure 2J, Table 1), but an intermediate (++) reaction in the acini and system ducts of the tapir’s parotid gland (Figure 2I, Table 1).

### 3.2. Mandibular Gland Histology and Histochemistry

**Figure 3:**
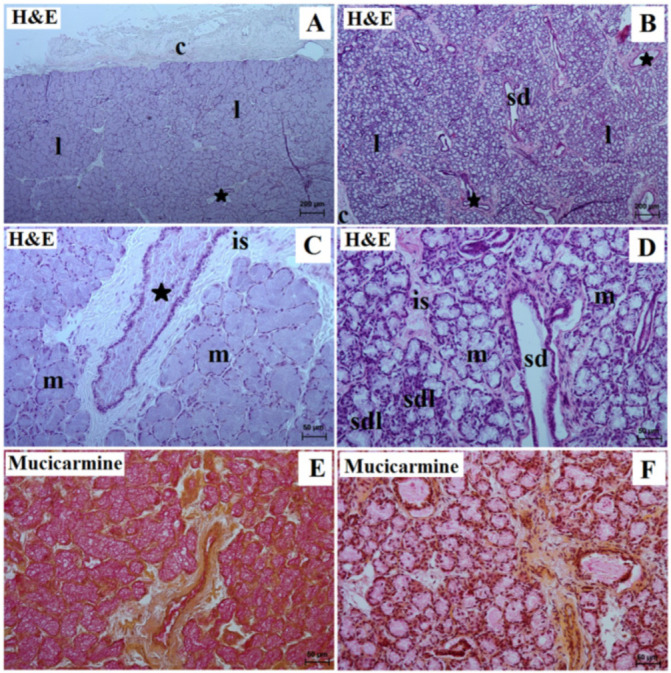
Photomicrograph of the lowland tapir (**A**,**C**,**E**) and aardvark (**B**,**D**,**F**) mandibular gland showing mucous acini (m) in the lowland tapir (**A**,**C**) and mixed acini with serous demilunes in the aardvark (**B**,**D**). The mucicarmine stain confirms a strongly positive reaction (+++) of the lowland tapir mucous acini and a slight reaction (+) in the mucous acini and middle (++) reaction in the serous demilunes in the aardvark. c—capsula, black asterisk—excretory interlobar ducts, is—interlobular septa, l—lobes, sd—striated ducts. Bar = 200 µm (**A**,**B**); 50 µm (**C**–**F**).

In both animals, the mandibular gland had a thick lenticular capsule with thin interlobar septa that penetrate deep into the gland, dividing the stroma into very large lobes (Figure 3A,B, Table 1). The duct system was well developed in the aardvark but not in the tapir. In both the examined animals, the interlobar ducts were composed of single columnar epithelium with centrally located nuclei. 

In the lowland tapir, the mandibular gland was a branched tubular complex gland producing a mucous secretion. The secretory units demonstrated a basophilic cytoplasm with flattened nuclei located in the basal parts of the cells (Figure 3C, Table 1). The tubules consisted of tall conical cells with very small lumens. The conical-shaped cells were relatively uniform in shape. The mean outer diameter was 85.0 µm.

In the aardvark, these glands were branched tubular alveolar glands producing mucous–serous secretions (Figure 3D, Table 1). The mean diameter of the serous cells was 18.0 µm. They formed a cap (serous demilunes) at the bottom of the tubules lined with mucous cells. The mucous cells appeared to be low, pyramidal cells with a wide base and a mean outer diameter of 42.0 µm. The apical part of the cytoplasm had a light, frothy appearance due to the mucinogen granules being washed out. In the basal part of the cell, a narrow zone of basophilic cytoplasm surrounded the kidney-shaped nucleus. 

In addition, in the aardvark, high numbers of striated ducts were seen; these were composed of a simple cuboidal epithelium with basal striations and secretory cells located within them; however, very few were observed in the lowland tapir (Figure 3D, Table 1). Mucicarmine stain yielded a strong (+++) positive reaction in the mucous cells and duct system in the lowland tapir; however, in the aardvark, a slight reaction (+) was observed in the mucous tubules and ducts system, and a moderate (++) reaction in the serous demilunes (Figure 3E,F, Table 1).

**Figure 4:**
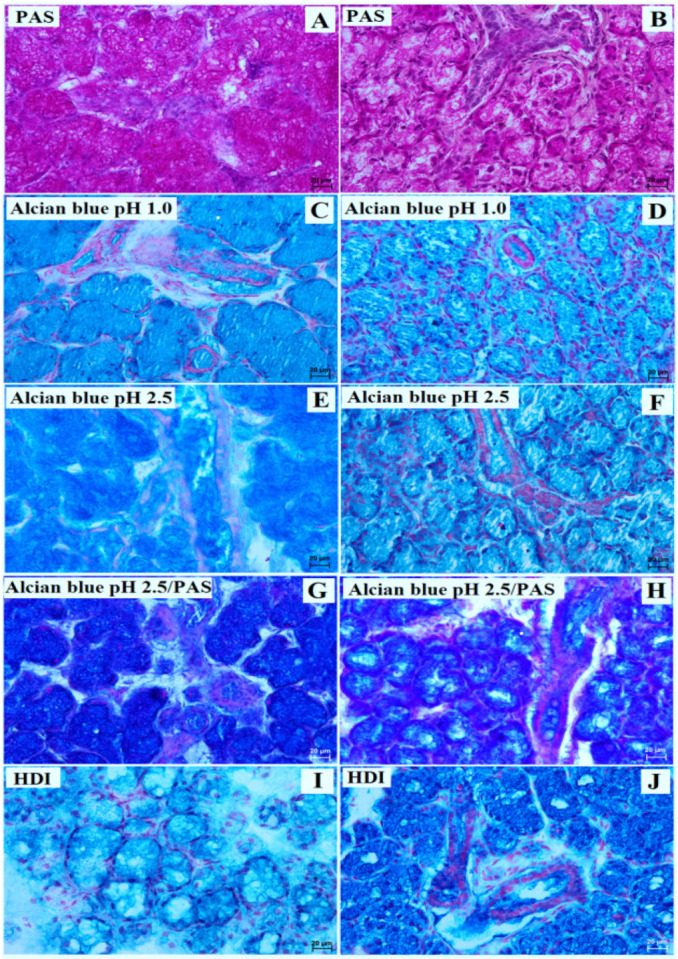
Photomicrograph of the lowland tapir (**A**,**C**,**E**,**G**,**I**) and aardvark (**B**,**D**,**F**,**H**,**J**) histochemistry mandibular gland. The secretion of the lowland tapir and aardvark was composed of PAS-positive neutral mucopolysaccharides, Alcian blue pH 1.0, Alcian blue pH 2.5, Alcian blue pH 2.5/PAS (blue color), and HDI-positive sulfated mucosubstances and sialomucins (**A**–**J**). Bar = 20 µm (**A**–**J**).

In both the examined animals, all histochemical studies, viz. PAS, Alcian blue pH 1.0, Alcian blue pH 2.5, Alcian blue pH 2.5/PAS (blue color), and HDI, demonstrated strong (+++) positive reactions in the secretory units, interlobar ducts, and striated ducts (Figure 4, Table 1).

## 4. Discussion and Conclusions

The histological structure of the individual salivary glands and the share of serous and mucous cells in their architecture varies depending on the diet and the environment in which the animal lives [18,19,20,21].

Histologically, the parotid gland in the examined lowland tapir consisted of a number of large lobes, which were formed by purely serous alveoli similar to humans, horses, donkeys, domestic ruminants, neonatal Indian buffalo, southern white-breasted hedgehogs, rats, rabbits, mice, African giant pouched rats, European hamsters, baboons, *Zaedyus pichiy*, and African palm squirrels [1,5,7,9,14,15,16,62,63,73,74,75,76,77,78]. Quinterelli and Dellovo [32] also report that the parotid gland was serous in tapirs, although they fail to indicate the species. However, while the gland lobes were formed by thick septa made of connective tissue in the previous study [32], they were formed by thin partitions in the present study; in addition, the gland lobes were ovoid in shape in [32] but were of different sizes and shapes in the present study.

In the Indian rhinoceros (*Rhinoceros unicornis*), another member of the Perissodactyla, H&E staining showed mucous-like granules in the parotid gland; in addition, DAB-based staining and TEM revealed the gland to have a bipartite structure with seromucous cells present [61]. Similarly, an interesting electron microscope study on the Mongolian gerbil (*Meriones meridianus*) [79] indicated that the nature of the discharge produced by the salivary glands is strongly influenced by the type of diet, as well as the habitat. 

Gaber et al. [80] found the secretion of the parotid gland in dogs to be serous, despite the fact that many studies have found it to be seromucous [1,81,82,83]. Gaber et al. [80] attribute this variation to diet; dry food causes the production of serous secretion, but a meat diet results in much more mucous. 

Our present findings indicate that the mandibular gland in the examined lowland tapir was divided into very large lobes consisting of only mucous tubules by poorly marked septa consisting of connective tissues. However, Quinterelli and Dellovo [32] report the gland to be a mucoserous type in tapirs (no species specified), in which mucous units dominate and few well-developed serous demilunes were present; however, the authors fail to note the size and shape of the lobes. In both the present study and the study by Quinterelli and Dellovo, the mucous cells demonstrate a basophilic cytoplasm. More significantly, however, the glandular duct system was found to be extremely well developed in Quinterelli and Dellovo [32] compared to the lowland tapir in the present study. 

The secretion of the mandibular gl”nd i’ also mucoserous in horses, hedgehogs, barking deer, and European hamsters [1,9,63,74,84]. However, in a *Rhinoceros* specimen, the mandibular gland was a mixed type but predominantly serous in nature, with the mucous tubules being restricted to discrete islands scattered throughout the obtrusively serous acini [59]; in addition, similarly to our lowland tapir, the gland was notably compact, with thin interlobar septa. In contrast, *Ceratotherium* and *Diceros* specimens demonstrated mandibular glands with a mixed character in which the mucous type dominated, with scattered small serous demilunes [59]. Such differentiation in the dominance of one secretory cell over another between rhino species further indicates that both diet and environment influence the structure and type of secretions of the major salivary glands; *Ceratotherium* and *Diceros* live in South and East Africa, whereas *Rhinoceros* live in Indie, Nepal, Indonesia, and Vietnam [85,86]. 

A few myrmecophagous mammals (aardvark, anteaters, armadillos, and pangolins), noted by Delsuc et al. [87], demonstrate similar but convergent morphological adaptations to their diet and their method of obtaining it, including sharp, powerful claws adapted to digging up mounds of ants and termites, reduced or absent teeth, elongated viscera, and a long, narrow tongue; they also demonstrate an overgrown major salivary gland with increased production of sticky saliva to obtain insects. 

In our examined aardvark, the parotid gland consisted of numerous small lobes divided by thick septa made of connective tissues. The lobes were composed of purely serous acini with strongly eosinophilic cytoplasm. However, Quinterelli and Dellovo [32] report the presence of basophilic cytoplasm in the serous acini of aardvarks. Serous type parotid glands were also observed in *Zaedyus pichiy* [73], *Chaetophractus villosus* [45], *Chaetophractus vellerosus* [73], *Dasypus hybridus* [43], *Dasypus novemcinctus* [53,88], *Cabassous loricatus* [88], and *Tachyglossus aculeatus* [47]. Dalguest and Werner [44] indicate the gland to be of a mucous nature in *Tamandua tetradactyla*, although seromucous cells were present in the parotid gland in the Malayan pangolin (*Manis javanica*) [51], and serous or mixed-type parotid glands were observed in *Proteles cristatus* [42]. 

The mandibular gland in our examined aardvark was composed of very large lobes and was classical a mucoserous type; however, Quinterelli and Dellovo [32] report these glands to be rather uniform and mucous type. Purely mucous glands have also been observed in *Tamandua tetradacyla* [52], *Tachyglossus aculeatus* [47], and *Manis javanica* [50], whereas seromucous glands were observed in *Myrmecophaga tridactyla* [49] and *Proteles cristatus* [40,41,42]. 

Interestingly, the mandibular gland in *Zaedyus pichiy* is characterized by two types of lobes: an anterior part with a mixed and serous character, and a posterior lobe with predominantly mucous units and some small serous acini [73]. Similar constructions were noted in species of Xenarthra: *Chaetophractus villosus* [45], *Chaetophractus vellerosus* [45], *Dasypus hybridus* [43], *Dasypus novemcinctus* [53,88], and *Cabassous loricatus* [88], which may be related to the feeding habitat of these animals. Allio et al. [89], Costa-Neto [90], Taylor and Frassaf [91], and Rahm [38] report the major salivary gland to be highly developed in the aardvark and describe it as hypertrophied. The gland produces a large amount of saliva which is used to extract prey from inside subterranean nests or termitaries, as well as to support the initial digestion of food in the mouth. 

In addition to allowing them to penetrate the nests of ants and termites, anteaters and pangolins also produce large amounts of sticky saliva to allow them to swallow their food whole, which is necessary due to their lack of teeth [92,93]. Amerongen et al., [94] and Tabak [95] propose that the glycoproteins present in the mandibular gland secretion play important roles associated with chemical defense, mechanical damages, microbial invasion in the oral cavity, transport of macromolecules for digestive efficiency, and prevention of proteolytic damage to the epithelium [54,55,56,57,96,97]. Kratzing and Woodall [98] also suggest that the mucous secretion in myrmecophagous animals protects the oral cavity and esophagus against terpenes secreted by termites, as observed in elephant shrews. However, Austin et al., [99] (cited by Anderson [42]) indicate that it is unknown whether salivary gland secretion may be involved in the detoxification of the terpene secretion of termites. 

Gooday [100] and Simunek et al. [101] (cited by Delsuc et al. [87]) suggest that the presence of chitinolytic bacteria in gut microbiomes in myrmecophagous mammals may influence the degradation of chitin exoskeletons to optimize protein intake. Studies based on comparative transcriptomic analysis of major salivary glands (chitinase gene expression) on 23 species of mammal, including anteaters and pangolins, found high expressions of CHIA paralogs, and indicate that the salivary glands may play a major role in allowing myrmecophagous and insectivorous mammals to adapt to an ant and termite diet [89]. It was also found that anteaters and pangolins differ in the repertoire of their chitinase (CHIA) genes, which affect the degradation of chitinous exoskeletons of ingested ants and termites [89]. The authors propose that the different chitinases found in anteaters and pangolins suggest the presence of divergent molecular mechanisms which may underpin convergent adaptation to an ant-eating diet in the studied myrmecophagous animals. 

Our study confirmed that partially the compositions of the glandular secretion of the parotid gland and mandibular gland of the lowland tapir were different than those in the aardvark. We were able to explain it mainly because of the diet. However, future analysis based on a higher number of animals should be performed. Additionally, the differences in the type of secretion of individual salivary glands related to age or even the differences between males and females cannot be excluded. 

## Figures and Tables

**Table 1:** Histological and histochemical characterization of the parotid and mandibular glands in the lowland tapir and aardvark.

	Lowland Tapir		Aardvark	
Items of Evaluation	Parotid Gland	Mandibular Gland	Parotid Gland	Mandibular Gland
Gland capsule	thin	thick	thin	thick
Interlobar septa	thin	thin	thick	thin
Lobes	different size (small, medium, large); various shapes (oval, elongated, triangular, quadrangular)	Very large	small and very numerous, ovoid-like shape	Very large
Type of gland	multilobed branched alveolar complex	multilobed branched tubular complex	multilobed branched alveolar complex	multilobed branched tubuloalveolar complex
Type of secretion	serous	mucous	serous	mucoserous
Secretory units	acini—single conical cells; average outer diameter—36.0 µm	tubules—tall conical cells with very small lumen, basophilic cytoplasm; average outer diameter—85.0 µm	acini—single conical cells; average outer diameter—25.0 µm	tubules—low pyramidal cells with a wide base and outer diameter average of 42.0 µm; acini (serous demilunes)—outer diameter average of 18.0 µm
Interlobar ducts	taller columnar cells with central or basally located oval or round nuclei; broad with wide lumina; very numerous	single columnar epithelium with centrally located nuclei; few	taller columnar cells with central or basally located oval or round nuclei; small with narrow lumina; few	single columnar epithelium with centrally located nuclei, well developed
Striated ducts	cuboidal or columnar cells with basal striations; many	simple cuboidal epithelium with basal striations; few	cuboidal or columnar cells with basal striations; rare	simple cuboidal epithelium with basal striations; very numerous
Mucicarmine stain	negative (−) reaction in the secretory units and ducts system	strong (+++) reaction in the mucous cells and ducts system	negative (−) reaction in the acini, striated ducts, and interlobar ducts	negative reaction (−) in the mucous units and ducts system, negative (−) reaction in the serous demilunes
PAS stain	negative (−) reaction in the acini; interlobar ducts with strong (+++) reaction; striated ducts with strong (+++) reaction	strong (+++) reaction in the tubules and all ducts system	strong (+++) reaction in the acini; interlobar ducts with strong (+++) reaction; striated ducts with slight (−/+) reaction	strong (+++) reaction in the tubules and all ducts system
Alcian blue pH 1.0 stain	acini, interlobar ducts, and striated ducts with negative (−) reaction	strong (+++) reaction in the tubules and all ducts system	acini, interlobar ducts, and striated ducts with negative (−) reaction	strong (+++) reaction in the tubules and all ducts system
Alcian blue pH 2.5 stain	acini, interlobar and striated ducts with strong (+++) reaction	strong (+++) reaction in the tubules and all ducts system	acini, interlobar and striated ducts with strong (+++) reaction	strong (+++) reaction in the tubules and all ducts system
Alcian blue pH 2.5/PAS stain	acini, interlobar and striated ducts with strong (+++) reaction—blue color	strong (+++) reaction in the tubules and all ducts system (blue color)	acini, interlobar and striated ducts with strong (+++) reaction—magenta color	strong (+++) reaction in the tubules and all ducts system (blue color)
HDI stain	middle (++) reaction in the acini and system ducts	strong (+++) reaction in the tubules and all ducts system	strong (+++) reaction in the acini and the system ducts	strong (+++) reaction in the tubules and all ducts system

## Data Availability

Material available upon request to the corresponding authors (joanna.kleckowska-nawrot@upwr.edu.pl; karolina.gozdziewska-harlajczuk@upwr.edu.pl).
